# N-linked glycosylation at Asn152 on CD147 affects protein folding and stability: promoting tumour metastasis in hepatocellular carcinoma

**DOI:** 10.1038/srep35210

**Published:** 2016-11-21

**Authors:** Jiang-Hua Li, Wan Huang, Peng Lin, Bo Wu, Zhi-Guang Fu, Hao-Miao Shen, Lin Jing, Zhen-Yu Liu, Yang Zhou, Yao Meng, Bao-Qing Xu, Zhi-Nan Chen, Jian-Li Jiang

**Affiliations:** 1 National Translational Science Center for Molecular Medicine, Department of Cell Biology, Fourth Military Medical University, Xi’an 710032, China

## Abstract

Cluster of differentiation 147 (CD147), also known as extracellular matrix metalloproteinase inducer, is a transmembrane glycoprotein that mediates oncogenic processes partly through N-glycosylation modifications. N-glycosylation has been demonstrated to be instrumental for the regulation of CD147 function during malignant transformation. However, the role that site-specific glycosylation of CD147 plays in its defective function in hepatocellular carcinomacells needs to be determined. Here, we demonstrate that the modification of N-glycosylation at Asn152 on CD147 strongly promotes hepatocellular carcinoma (HCC) invasion and migration. After the removal of N-glycans at Asn152, CD147 was more susceptible to degradation by ER-localized ubiquitin ligase-mediated endoplasmic reticulum-associated degradation (ERAD). Furthermore, N-linked glycans at Asn152 were required for CD147 to acquire and maintain proper folding in the ER. Moreover, N-linked glycans at Asn152 functioned as a recognition motif that was directly mediated by the CNX quality control system. Two phases in the retention-based ER chaperones system drove ER-localized CD147 trafficking to degradation. Deletion of N-linked glycosylation at Asn152 on CD147 significantly suppressed *in situ* tumour metastasis. These data could potentially shed light on the molecular regulation of CD147 through glycosylation and provide a valuable means of developing drugs that target N-glycans at Asn152 on CD147.

Alterations in the glycans of glycoproteins are involved in cell signalling and communication, tumour cell dissociation and invasion, tumour angiogenesis, immune modulation and metastasis formation^1^,^2^,^3^,^4^. During malignant transformation, many glycoproteins undergo a wide range of glycosylation alterations with implications for biological function in different cell populations^5^. For example, abnormal alterations in the glycosylation profile of E-cadherin affects its cellular localization, molecular assembly, stability of adherens junctions, and cell–cell aggregation^6^,^7^. There is increasing evidence to indicate that protein-linked glycans, which provide additional recognition epitopes for protein receptors, also depend on the precise location of N-glycosylation sites because not all sites are equally important^8^,^9^,^10^. Among the nine N-glycosylation sites, mutation of Asn548 reduces the interaction between CD133 and β-catenin and significantly inhibits the ability of CD133 to promote hepatoma cell growth^11^. The role of glycans in the underlying mechanisms of various cancers has been highlighted by the fact that glycan alterations at specific glycosylation sites regulate the development and progression of cancer, serving as important biomarkers and providing a set of valuable targets for diagnosing cancer and developing novel therapeutic strategies against carcinomas^12^,^13^,^14^,^15^.

Cluster of differentiation 147 (CD147), which is considered to be a cancer-associated biomarker for pathological diagnoses, prognostic evaluations, and targeted therapies, is a transmembrane protein that is highly expressed in various cancers^16^,^17^. The induction of MMPs secretion is an important function of CD147, significantly promoting tumour cell invasion and metastasis^18^. Several mechanisms have been proposed to explain the regulation of CD147 expression, including the following mechanisms: the TGF-β1-CD147 signalling loop in the development of liver fibrosis; the regulatory loop involving miR-22, Sp1, and c-Myc modulating CD147 expression; and the induction of CD147 clustering by galectin-3^19^,^20^,^21^. Among the various mechanisms, the modification of CD147 by N-glycosylation has been demonstrated to be instrumental for the regulation of CD147 function during malignant transformation^22^. Previous studies have suggested that CD147 deglycosylation by tunicamycin treatment not only results in a failure to induce MMP-1 and MMP-2 expression, but also inhibits the induction of MMPs secretion caused by native CD147^23^. A study in our laboratory that resolved the crystal structure of CD147 identified three N-linked glycosylation sites on CD147: Asn44, Asn152 and Asn186^24^,^25^. Depending on the location, conformation and even the physiopathological context of the acceptor tripeptide, some N-glycosylation sites are more important than others in determining the protein’s function. However, the key N-glycosylation site(s) of CD147 that may be critical for regulatingits functions in HCC remain to be determined.

In the present study, we revealed that the modification of N-glycosylation at Asn152 is required for the function of CD147. After the removal of N-glycans at Asn152, CD147 is degraded partly by ER-localized ubiquitin ligase-mediated ERAD. Furthermore, N-glycans at Asn152 directly interact with the CNX-mediated quality control system. Last but not least, the deletion of N-linked glycosylation at Asn152 on CD147 significantly suppressed *in situ* tumour metastasis.

## Results

### Modifications of N-glycosylation at Asn152 on CD147 promotes HCC cell invasion and migration

**Figure 1:**
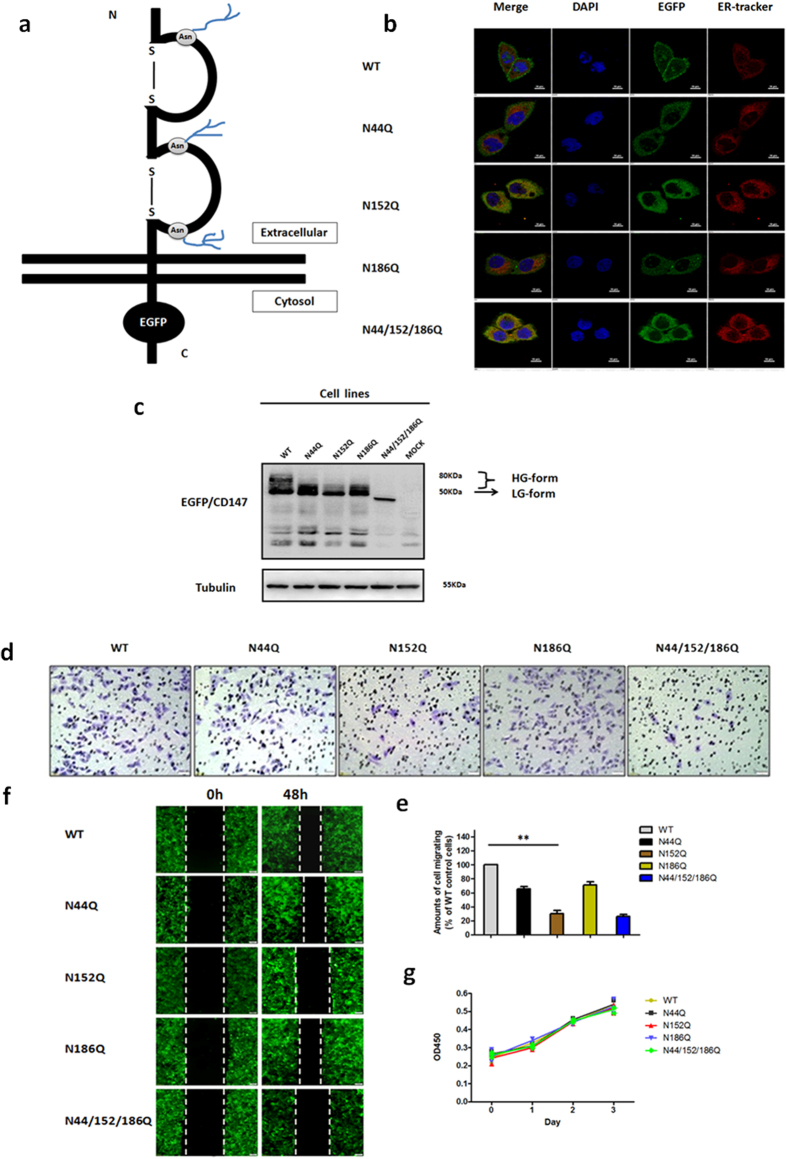
N-glycans at Asn152 are required for CD147 function. (**a**) Model of the CD147 N-glycosylation site. The positions of each mutation in CD147 are illustrated. (**b**) Localization of CD147-EGFP. K7721 cells stably expressing wild-type or mutant CD147-EGFP (green) were immunostained using an ER-Tracker (red) and DAPI (blue) and observed using confocal laser scanning microscopy. Scale bars, 20 μm. (**c**) Expression of the CD147-EGFP. Cell lysates of K7721 cells stably expressing WT and each mutant were analysed by Western blotting using an anti-EGFP antibody. (**d,e**) Invasive potential of each cell line. Representative phase contrast image analysis and a quantitative analysis of the transwell invasive potential assay in each cell line. Values indicate the mean ± standard error of the mean (SEM) of three independent experiments. **P < 0.01. (**f**) Migration ability of each cell line. Representative phase contrast image analysis for the wound healing assay in each cell line. (**g**) Proliferation of each cell line. An *in vitro* cell counting kit (CCK)-8 assay was used to examine cell growth based on absorbance at 450 nm at the indicated time.

To better understand the significance of N-glycans at specific glycosylation site in regulating the function of CD147, we constructed CD147-knockout SMMC-7721 HCC cell lines (K7721) stably expressing either the WT or single-site glycosylation mutations (Fig. 1a). Immunofluorescence staining assays showed that CD147(WT)-EGFP was primarily localized to the plasma membrane (Fig. 1b). By contrast, CD147(N152Q)-EGFP and CD147(N44/152/186Q)-EGFP were detected in a reticular pattern that largely colocalized with the ER-tracker (Fig. 1b), indicating that they were retained in the ER. Other mutants were observed at both the cell-surface and with intracellular localizations (Fig. 1b). Next, we characterized the CD147-EGFP proteins using immunoblotting with an anti-EGFP antibody. As shown in Fig. 1c, CD147(WT)-EGFP migrated with two separate bands at approximately 50–80 kDa, indicating that it was present as a post-ER form (highly glycosylated form: HG-CD147). CD147(N152Q)-EGFP and CD147(N44/152/186Q)-EGFP were detected as a sharp band at approximately 50 kDa, which is thought to represent the ER form (high-mannose form: LG-CD147). CD147(N44Q)-EGFP and CD147(N186Q)-EGFP were largely detected as the ER form, but a fraction of the pool of mutant proteins were migrated to the position of post-ER form, as shown by a diffuse band. Moreover, the CD147(N152Q)-EGFP and CD147(N44/152/186Q)-EGFP proteins were present at a low level in the total cell lysates compared with the levels of CD147(WT)-EGFP. These results indicate that, among the three sites, N-glycosylation at Asn152 might be the most critical for the biological function of CD147.

To detect whether CD147-induced tumour cell malignant transformation was mediated by modifications of the N-glycosylation at Asn152 in HCC cells, transwell migration and wound healing assays were performed. As shown in Fig. 1d–f, the removal of N-glycans at Asn152 markedly reduced cell invasion and migration. However, wild-type CD147 and individual N-glycosylation mutants had no effect on cell proliferation, as assessed using the cell counting kit (CCK)-8 assay (Fig. 1g).

Collectively, these data demonstrate that modifications of N-glycosylation at Asn152 on CD147 play crucial roles in promoting HCC cell invasion and migration.

### After the removal of N-glycans at Asn152, CD147 is degraded partly by ER-localized ubiquitin ligase-mediated ERAD

**Figure 2:**
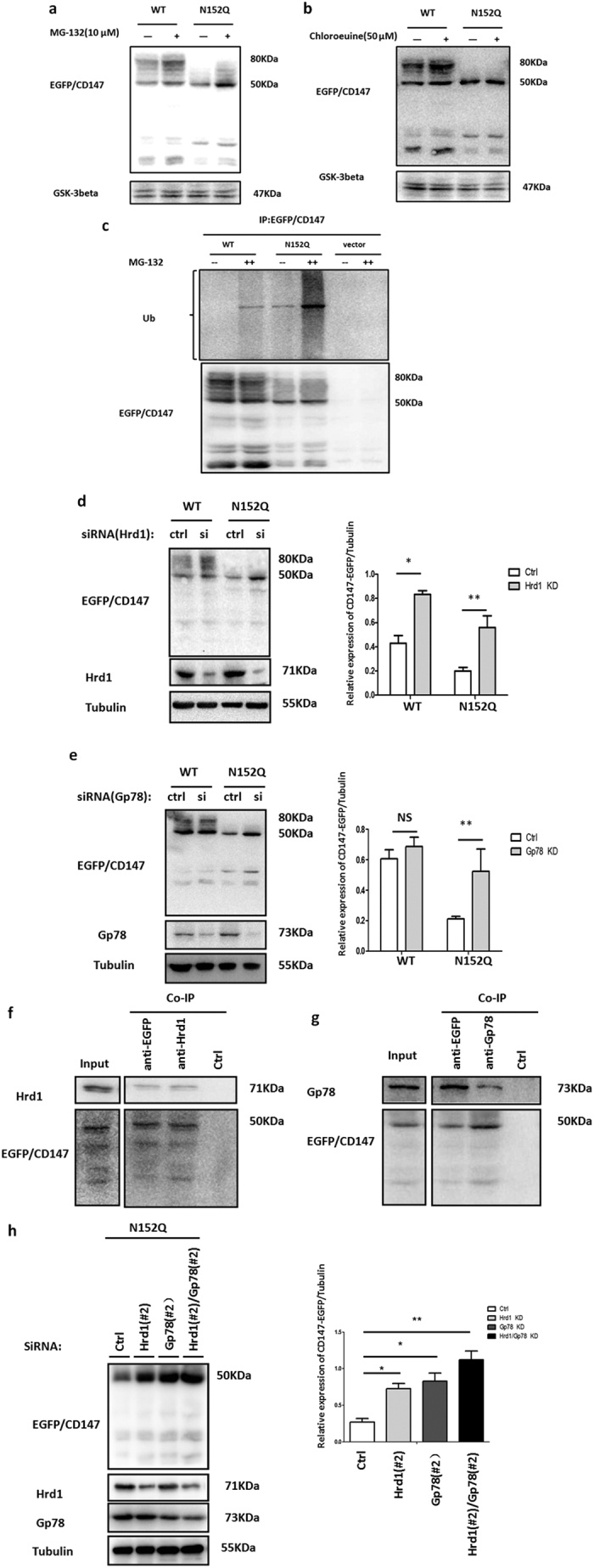
E3 ubiquitin ligases Hrd1 and Gp78 were involved in the degradation of CD147(N152Q)-EGFP. (**a**) The effect of proteasome inhibitor (MG-132) on CD147(WT)-EGFP and CD147(N152Q)-EGFP. K7721 cells stably expressing CD147(WT)-EGFP and CD147(N152Q)-EGFP were cultured for 24 h in the presence or absence of 10 μM MG-132. The cell lysates were immunoblotted with the indicated antibodies. (**b**) The effect of lysosomal inhibitor (chloroquine) on the CD147(WT)-EGFP and CD147(N152Q)-EGFP. K7721 cells stably expressing CD147(WT)-EGFP and CD147(N152Q)-EGFP were cultured for 16 h in the presence or absence of 50 μM chloroquine. The immunoblots of cell lysates were probed with the indicated antibodies. (**c**) Both CD147(WT)-EGFP and CD147(N152Q)-EGFP were ubiquitinated. Extracts prepared from K7721 cells stably expressing CD147(WT)-EGFP or CD147(N152Q)-EGFP were immunoprecipitated (IP) using an anti-EGFP antibody, and the resulting precipitates were examined by immunoblot analysis using the indicated antibodies. (**d**) Knockdown of Hrd1 increased the protein levels of CD147(WT)-EGFP and CD147(N152Q)-EGFP. K7721 cells stably expressing CD147(WT)-EGFP or CD147(N152Q)-EGFP were transfected with control or Hrd1 siRNAs for 3 days. Immunoblots of cell lysates were probed with the indicated antibodies. Relative expression of CD147-EGFP/Tubulin were quantified using Image J software, *P < 0.05, **P < 0.01. (**e**) Knockdown of Gp78 increased the protein level of CD147(N152Q)-EGFP. K7721 cells stably expressing CD147(WT)-EGFP or CD147(N152Q)-EGFP were transfected with control or Gp78 siRNA for 3 days. Immunoblots of cell lysates were probed with the indicated antibodies. Relative expression of CD147-EGFP/Tubulin were quantified using Image J software, no statistical significance (NS), **P < 0.01. (**f,g**) CD147(N152Q)-EGFP co-immunoprecipitated with Hrd1 and Gp78. Extracts prepared from K7721 cells stably expressing CD147(N152Q)-EGFP were coimmunoprecipitated using the indicated antibodies. The resulting precipitates were examined by immunoblot analysis using the indicated antibodies. (**h**) Additive effect of Hrd1 and Gp78 co-inactivation on CD147(N152Q)-EGFP. K7721 cells stably expressing CD147(N152Q)-EGFP were transfected with control siRNA, or siRNA against Hrd1 (#2), Gp78 (#2), or both for 3 days. Lysates prepared from these cells were immunoblotted using the indicated antibodies. Relative expression of CD147-EGFP/Tubulin were quantified using Image J software, *P < 0.05, **P < 0.01.

To examine the effect of N-glycans at Asn152 on CD147 stability, we detected the degradability of CD147. Given the possibility of differences in the levels of mRNA derived from the cell lines, we first determined the CD147 mRNA level. Real-time quantitative PCR analysis showed no significant differences among the cell lines (Fig. S1a). Because the substrates of the ERAD system are degraded by the proteasome system, we treated cells with the proteasome inhibitor MG-132 and detected the accumulation of the ER and post-ER forms of the CD147(WT)-EGFP and CD147(N152Q)-EGFP proteins (Fig. 2a). Treating cells with Chloroquine, which inhibited the lysosome system pathway by inhibiting the activity of protease in the lysosome, increased the levelof the post-ER form of CD147(WT)-EGFP, indicating that part of the CD147(WT)-EGFP was transported into lysosomes and degraded. However, Chloroquine treatment did not alter the levels of CD147(N152Q)-EGFP (Fig. 2b). Ubiquitination experiments further demonstrated that the loss of N-glycans at Asn152 led to a pronounced increase in the ubiquitination of CD147 in the presence of MG-132 (Fig. 2c), suggesting that a portion of the CD147(N152Q)-EGFP mutant that accumulated in the ER was degraded in a primarily proteasome-dependent manner.

The mammalian ERAD network is organized around two key ubiquitin ligases, Hrd1 and Gp78. To determine whether CD147 was recognized and degraded by ER-localized ubiquitin ligases, we treated cells stably expressing CD147(WT)-EGFP and CD147(N152Q)-EGFP with small interfering RNA(siRNA) against Hrd1 and Gp78. The knockdown of Hrd1 resulted in increased total protein levels in the WT and N152Q cells (Fig. 2d), suggesting that Hrd1 is required for the degradation of ER-localized CD147. The knockdown of Gp78 only increased the level of CD147 in the N152Q cells, indicating that Gp78 is also responsible for the degradation of CD147(N152Q)-EGFP (Fig. 2e). Furthermore, we observed that the expression of Hrd1 and Gp78 were up-regulated in N152Q cells compared with their expression in WT cells (Fig. 2d,e, Fig. S1b,c).

We also analysed the interaction of the ubiquitin ligases with the ER-localized form of CD147(N152Q)-EGFP through co-immunoprecipitation experiments. We found that both Hrd1 and Gp78 strongly interacted with CD147(N152Q)-EGFP (Fig. 2f,g). Because both Hrd1 and Gp78 were directly involved in the degradation of ER-localized CD147, we next examined the effect of the knockdown of both Hrd1 and Gp78 on the CD147(N152Q)-EGFP mutant. The level of CD147(N152Q)-EGFP was additively increased when both Hrd1 and Gp78 were knocked down (Fig. 2h), suggesting that Hrd1 and Gp78 individually mediate the degradation of CD147(N152Q)-EGFP. These results suggest that bulky hydrophilic N-glycans at Asn152 stabilize the proper tertiary structure of CD147 to protect against degradation mediated byHrd1 and Gp78 in the ER.

### N-glycosylation at Asn152 is required for CD147 to acquire and maintain its proper folding system in the ER

**Figure 3:**
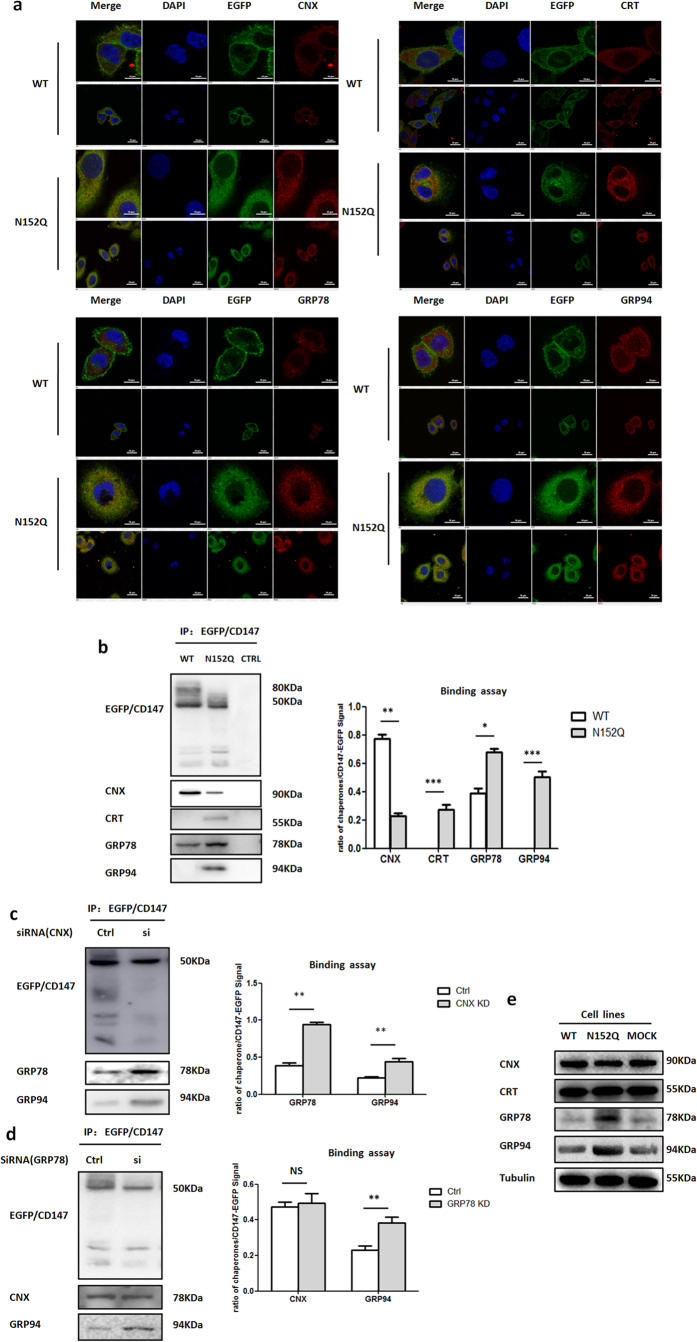
CD147(N152Q)-EGFP was more associated with the heat shock proteins than with the ER lectin chaperones. (**a**) The localizations of CD147-EGFP and its binding partners, CNX, CRT, GRP78, and GRP94, were determined by immunofluorescence staining in K7721 cells stably expressing CD147(WT)-EGFP and CD147(N152Q)-EGFP. Scale bars, 20 μm and 10 μm. (**b**) The interaction between CD147 and the chaperones. Extracts prepared from K7721 cells stably expressing CD147(WT)-EGFP or CD147(N152Q)-EGFP were immunoprecipitated using the indicated antibodies. The resulting precipitates were examined by immunoblot analysis using the indicated antibodies. The signal intensities of each chaperone and CD147-EGFP were quantified using Image J software and are presented as the ratio of chaperone/CD147-EGFP signal. *P < 0.05, **P < 0.01, and **P < 0.001. (**c**) The effect of CNX knockdown on the binding ability among CD147(N152Q)-EGFP, GRP78, and GRP94. K7721 cells stably expressing CD147(N152Q)-EGFP were transfected with control or CNX siRNA for 3 days, followed by immunoprecipitated with the indicated antibodies, **P < 0.01. (**d**) The effect of GRP78 knockdown on the binding ability among CD147(N152Q)-EGFP, CNX, and GRP94. K7721 cells stably expressing CD147(N152Q)-EGFP were transfected with control or CNX siRNAs for 3 days, followed by immunoprecipitated with the indicated antibodies, **P < 0.01. (**e**) Expression of CNX, CRT, GRP78, and GRP94. Lysates of K7721 cells stably expressing the CD147(WT)-EGFP and CD147(N152Q)-EGFP mutants were analysed by Western blotting with the indicated antibodies.

The above results show that after mutation of N-glycosylation at Asn152, CD147 was identified as a misfolded or unfolded protein that was recognized and destroyed via the ER-associated degradation pathway. We predicted that the N-glycosylation site Asn152, which is located at the middle of the Ig-V domain on CD147, would disturb the interactions between CD147 and the partner proteins that assist in protein folding, trafficking, and/or expression^22^,^24^. In screening the CD147(N152Q)-EGFP binding partners, we used cross-linking, IP and an LC-MS/MS analysis to identify the partners that interacted with CD147. We selectively identified the following CD147-EGFP interacting proteins in the ER: the heat shock proteins GRP78 and GRP94 and the ER lectin chaperones calnexin (CNX) and calreticulin (CRT) (Table S1). Immunofluorescence staining assays showed that a loss of N-glycans at Asn152 resulted in an increased recruitment of chaperones that colocalized with CD147 (Fig. 3a). Immunoprecipitation experiments also verified the presence of interactions between CD147 and these chaperones (Fig. 3b). Furthermore, we found that two lectin chaperones showed opposite binding capacities towards the mutant CD147(N152Q)-EGFP. The interaction between CD147(WT)-EGFP and CNX was largely dependent on N-glycans at Asn152, whereas CRT interacted more strongly with CD147(N152Q)-EGFP mutants (Fig. 3b). By contrast, the heat shock proteins presented a consistent trend; GRP78 and GRP94 interacted more strongly with CD147(N152Q)-EGFP than with CD147(WT)-EGFP (Fig. 3b).

Based on the above results, we hypothesized that CD147 proteins without N-glycans at Asn152, which appear to adopt an alternative conformation that is distinct from the WT protein, rely more strongly on the heat shock proteins than on the ER lectins. To test this hypothesis, we treated K7721 cells stably expressing CD147(N152Q)-EGFP with small interfering RNA (siRNA) against CNX or GRP78 and measured the binding capacity of CD147. The knockdown of CNX resulted in strong interactions between the CD147(N152Q)-EGFP mutant and the heat shock proteins GRP78 and GRP94, whereas the knockdown of GRP78 markedly enhanced the binding of CD147 to GRP94 relative to its binding to CNX (Fig. 3c,d and Fig. S2a,b). Consistent with the above findings, we also found that the abnormal accumulationof the CD147(N152Q)-EGFP induced ER stress and that unfolded protein response (UPR) was activated in the N152Q cells (Fig. S2c.d). Under these conditions, Western blotting showed that the heat shock proteins GRP78 and GRP94 were up-regulated, and no changes in the lectin chaperones were observed in the N152Q cells (Fig. 3e and Fig. S2e). Taken together, it appears that N-linked glycosylation at Asn152 is critical for CD147 to acquire and maintain its correct folding pattern in the ER.

### N-Glycans at Asn152 are required for CD147 release from the CNX quality control system

**Figure 4:**
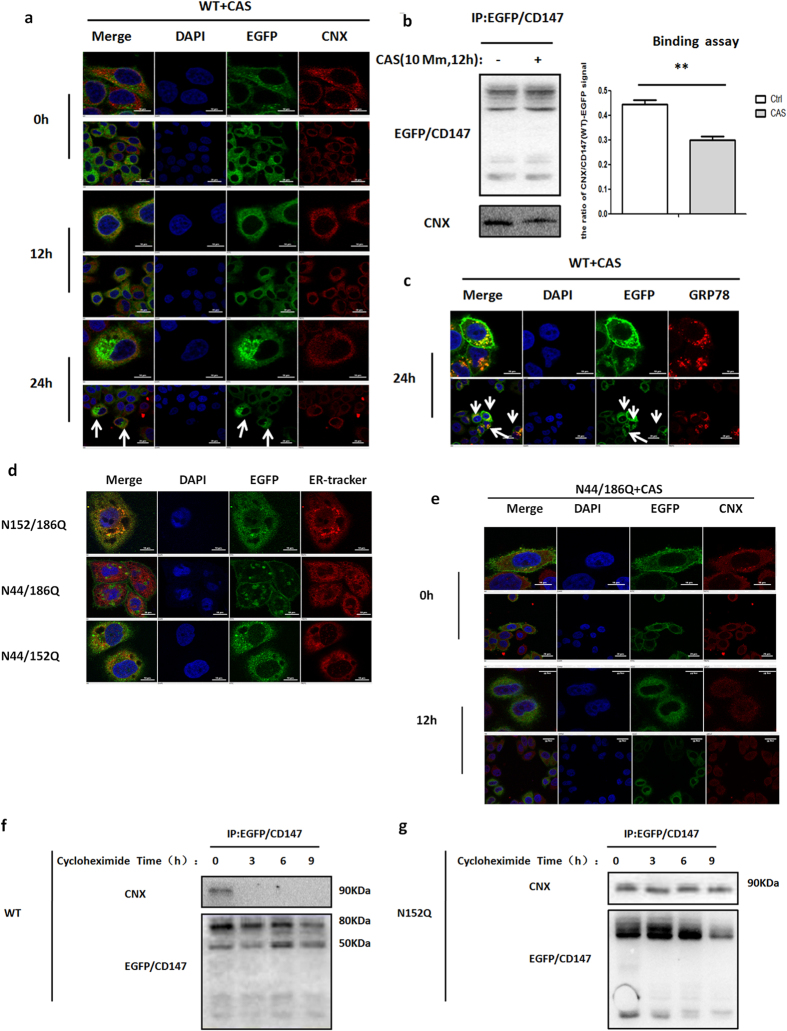
N-glycans at Asn152 are required for CD147 release from the CNX quality control system. (**a**) The localizations of CD147(WT)-EGFP and CNX. Incubation of K7721 cells stably expressing CD147(WT)-EGFP with 1 mM CAS for the indicated time. CNX was analysed by immunofluorescence staining. Scale bars, 20 μm and 10 μm. (**b**) The binding of CD147 with CNX. The interaction between CD147(WT)-EGFP and CNX after a 12-h incubation of K7721 cells stably expressing CD147(WT)-EGFP in the presence of 1 mM CAS. **P < 0.01. (**c**) The localizations of CD147(WT)-EGFP and GRP78. Incubation of K7721 cells stably expressing CD147(WT)-EGFP with 1 mM CAS for the indicated time. GRP78 was analysed by immunofluorescence staining. Scale bars, 20 μm and 10 μm. (**d**) Localization of CD147-EGFP. K7721 cells transiently expressing mutant CD147(N152/186Q)-EGFP, CD147(N152/186Q)-EGFP, and CD147(N152/186Q)-EGFP (green) were immunostained using an ER-Tracker (red) and DAPI (blue) and observed using a confocal laser scanning microscopy. Scale bars, 20 μm. (**e**) The localizations of CD147(N44/186Q)-EGFP and CNX. Incubation of K7721 cells transiently expressing CD147(N44/186Q)-EGFP with 1 mM CAS for the indicated time, CNX was analysed by immunofluorescence staining. Scale bars, 20 μm and 10 μm. (**f,g**) CD147(WT)-EGFP or CD147(N152Q)-EGFP was immunoprecipitated before or after the incubation of K7721 cells stably expressing CD147(WT)-EGFP or CD147(N152Q)-EGFP with 20 μg/mL cycloheximide for the indicated periods of time.

Lectin chaperones can utilize the N-glycans of a glycoprotein as a quality control signal to guide the newly synthesized proteins through highly ordered processing and folding steps^26^,^27^,^28^. We next determined whether N-glycans at Asn152 function as a recognition motif to assist CD147 release from the CNX quality control system. Because CNX binds to nascent glycoproteins only after the progressive removal of two glucose residues from N-glycans by ER glucosidases I and II, castanospermine (CAS) prevents the interaction between N-glycans and CNX^29^,^30^. As shown in Fig. 4a, compared with the result in the absence of CAS, the incubation of K7721 cells stably expressing CD147(WT)-EGFP with CAS for 12 h resulted in an overlap between CD147 and CNX, indicating that N-glycans are required for the folding and maturation of CD147 via an interaction with CNX. Immunoprecipitation experiments were also performed to test whether a 12-h incubation with CAS inhibited the interaction between the CNX and CD147. The data showed that N-glycans enhanced the interaction between CD147 and CNX, which was blocked in the presence of CAS (Fig. 4b). Notably, the incubation of K7721 cells stably expressing CD147(WT)-EGFP with CAS for 24 h resulted in protein aggregation within the cells (Fig. 4c), suggesting that proteins could not refold into their native conformation. We also found that GRP78 spontaneously accumulated in areas where CD147 aggregated (Fig. 4c). Based on the above results, we concluded that N-glycans are essential for the appropriate release of CD147 from the CNX quality control system.

Next, we tested whether N-glycans at Asn152 serve as a recognition motif that directly mediate the CNX quality control system. We applied site-directed mutagenesis to generate double-site glycosylation mutations. Immunofluorescence showed that CD147(N44/186Q)-EGFP, which had a modification of N-glycosylation at Asn152, was present on the cell surface, whereas CD147(N44/152Q)-EGFP and CD147(N152/186Q)-EGFP were retained in the ER compartments (Fig. 4d). Similarly, CD147(N44/186Q)-EGFP accumulated in the ER after a 12-h CAS treatment (Fig. 4e). Furthermore, CD147(WT)-EGFP binding to CNX was detected before but not after incubation of the cell with cycloheximide for 3 h (Fig. 4f). By contrast, the association of CD147(N152Q)-EGFP with CNX was retained in the ER even after incubation of the cells were incubated with cycloheximide for 9 h (Fig. 4g), indicating that after the removal of N-glycans at Asn152, CD147underwent repeated post-translational CNX-assisted folding attempts in the ER. These data demonstrate that the N-glycosylation site Asn152 on CD147 functions as a recognition motif that directly mediate the CNX quality control system.

### Two phases in the retention-based ER chaperones system drive ER-localized CD147 trafficking towards degradation

**Figure 5:**
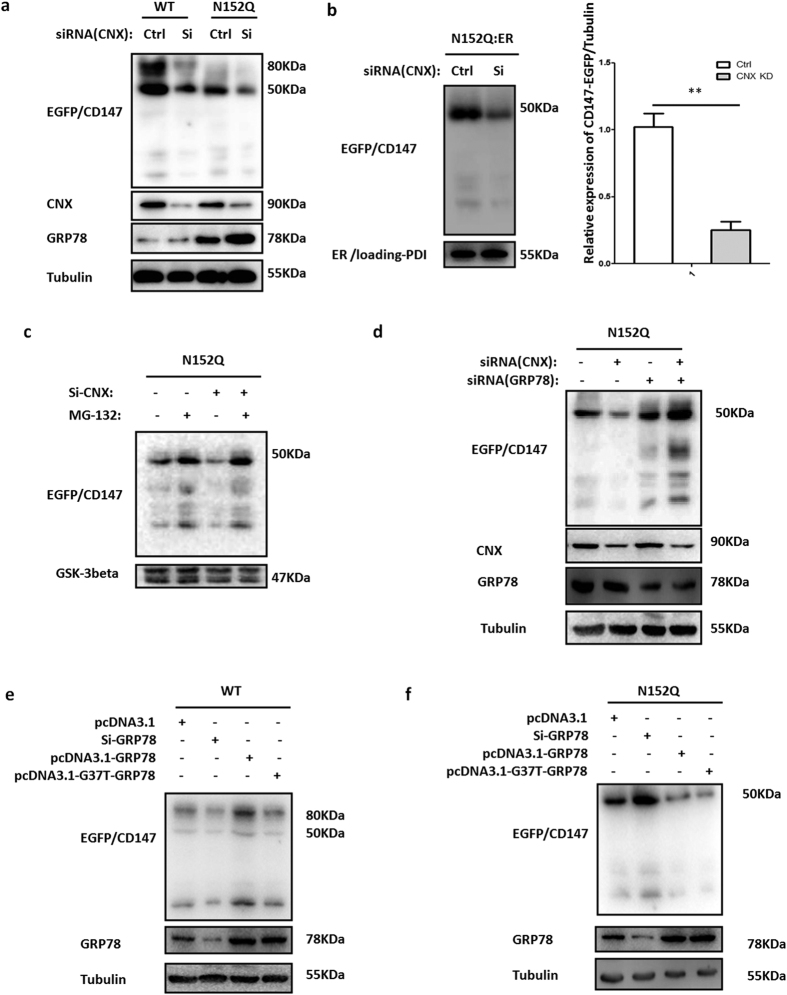
Progression of ER-localized CD147 correlated with its transfer from CNX to GRP78. (**a**) The effect of CNX knockdown on the expression of CD147(WT)-EGFP and CD147(N152Q)-EGFP. K7721 cells stably expressing CD147(WT)-EGFP or CD147(N152Q)-EGFP were transfected with control or CNX siRNA for 3 days. Immunoblots of cell lysates were probed with the indicated antibodies. (**b**) The effect of CNX knockdown on the expression of CD147(N152Q)-EGFP in the ER. K7721 cells stably expressing CD147(N152Q)-EGFP were transfected with control or CNX siRNA for 3 days. Then, the cells were through fractionating cells into ER using the ER isolation kit. Immunoblots of ER proteins were probed with the indicated antibodies. Relative expression of CD147-EGFP/Tubulin were quantified using Image J software, **P < 0.01. (**c**) The effect of CNX knockdown on the expression of CD147(N152Q)-EGFP in the presence of proteasome inhibitor (MG-132). K7721 cells stably expressing CD147(N152Q)-CD147 with the control or siRNA against CNX in the presence or absence of MG-132. Immunoblots of cell lysates were probed with the indicated antibodies. (**d**) Additive effect of CNX and GRP78 co-inactivation on CD147(N152Q)-EGFP. K7721 cells stably expressing CD147(N152Q)-EGFP were transfected with the control or siRNA against CNX, GRP78 or both for 3 days. Lysates prepared from these cells were immunoblotted using the indicated antibodies. (**e,f**) ATPase activity was not necessary for the degradation of CD147(N152Q)-EGFP. K7721 cells stably expressing CD147(WT)-EGFP or CD147(N152Q)-EGFP were transfected with control or GRP78 siRNA, wild-type GRP78 and mutant G37T-GRP78 for 3 days. The resulting precipitates were examined by immunoblot analysis using the indicated antibodies.

Based on our observation that CNX and GRP78 are involved in the retention of CD147 in the presence of CAS, we further tested whether CNX and GRP78 were involved in the degradation of CD147(N152Q)-EGFP. As show in Fig. 5a, the knockdown of CNX reduced the level of CD147(WT)-EGFP and CD147(N152Q)-EGFP. Notably, CNX knockdown led to an increased level of GRP78 in the N152Q cells compared with levels in the siRNA control groups, suggesting that GRP78 may be involved in the degradation of CD147(N152Q)-EGFP. Meanwhile, the knockdown of CNX reduced the expression of CD147(N152Q)-EGFP in the ER, suggesting that CD147(N152Q)-EGFP was transported out from the ER and degraded (Fig. 5b and Fig. S3a). We also treated K7721 cells stably expressing CD147(N152Q)-EGFP with control or siRNA against CNX in the presence or absence of the proteasome inhibitor MG-132, and we detected the accumulation of the ER form of CD147 (Fig. 5c and Fig. S3b). Meanwhile, the knockdown of both CNX and GRP78 additively delayed the degradation of CD147(N152Q)-EGFP, suggesting that CNX and GRP78 cooperatively function in the early stage of CD147(N152Q)-EGFP degradation (Fig. 5d). We also found that GRP78 promoted the expression of CD147(WT)-EGFP in the WT cells, which was dependent on ATPase (Fig. 5e), whereas GRP78 mediated the degradation of CD147 independent of ATPase in N152Q cells(Fig. 5f). These results implied that ER-localized CD147 trafficked to degradation via the two phases in the retention-based ER chaperone is based on the effect of N-linked glycosylation at Asn152 on the intracellular instability of CD147.

### Deletion of N-linked glycosylation at Asn152 on CD147 significantly suppressed *in situ* tumour metastasis

**Figure 6:**
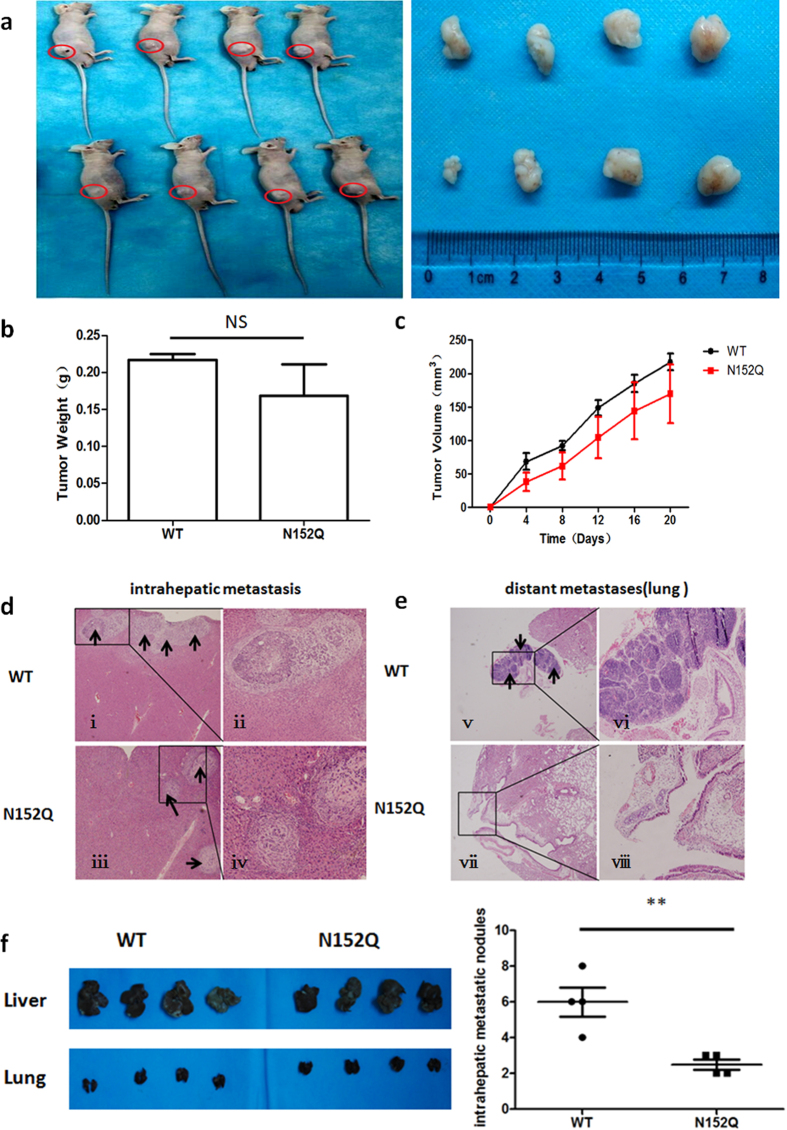
Effects of N-glycans at Asn152 on subcutaneous tumours and orthotopic liver tumours. (**a–c**) Subcutaneous tumour weights and growth curves. *In vivo* subcutaneous tumour weights and growth curves for CD147(WT)-EGFP or CD147(N152Q)-EGFP cells (n = 4). (**d,e**) N-glycans at Asn152 inhibited HCC cell invasion and metastasis *in vivo*. Haematoxylin-eosin-stained sections of intrahepatic metastatic nodules and distant metastatic nodules in the lung formed by K7721 cells stably expressing CD147(WT)-EGFP or CD147(N152Q)-EGFP 8 week after injection. Black arrows indicate the metastatic foci in the liver or lung tissure. Magnification: i, ii, v, and vi, ×100; iii, iv, vii, and viii, ×200. (**f**) The number of metastatic nodules. The numbers of metastatic nodules in each mouse liver were counted, **P < 0.01.

To further determine the effect of N-glycans at Asn152 on the oncogenicity of CD147 in HCC progression, K7721 cells stably expressing CD147(WT)-EGFP or CD147(N152Q)-EGFP were subcutaneously implanted into nude mice. The results showed that the removal of N-glycans at Asn152 on CD147 had no significant impact on overall tumour growth, as assessed based on tumour weights and tumour volume growth curves (Fig. 6a–c). Consistent with the above findings, an orthotopic tumour model yielded a similar tumour size at the original injection site (Fig. 6d). The effect of N-glycans at Asn152 on the *in vivo* metastasis model evaluated by the orthotopic liver was subsequently tested. K7721 cells stably expressing CD147(WT)-EGFP or CD147(N152Q)-EGFP were orthotopically injected into the livers of nude mice. The number of metastatic nodules in the liver dramatically decreased in mice injected with the K7721 cells that were stably expressing CD147(N152Q)-EGFP compared with the number in WT control injections (Fig. 6f and Fig. S4). With respect to metastatic spread into the lungs, none of the mice in the CD147(N152Q)-EGFP group showed distant lung metastases, whereas 2 of 4 mice in the WT group had lung metastases (Fig. 6e). Our data suggeste that N-glycans at Asn152 are a critical factor for CD147-promoted HCC cell metastasis.

## Discussion

**Figure 7:**
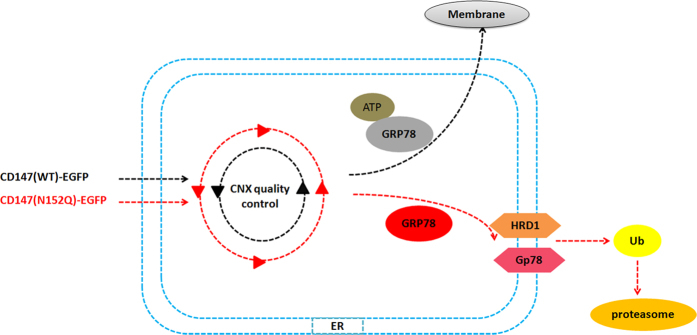
Schematic representation of the major molecular mechanisms of N-glycosylation at Asn152 in regulating the folding and stability of CD147 in HCC.

The functional activation or up-regulation of CD147 is considered to be a hallmark of tumour cell invasion and metastasis^31^,^32^. This study provides insight into the mechanisms by which the modification of N-glycosylation at Asn152 is instrumental in the regulation of CD147 function during malignant transformation (Fig. 7). We discovered that CD147 promotes HCC metastasis by modifying N-glycans at Asn152. The removal of N-glycans at Asn152 not only resulted in the loss of the glycan components on CD147 most closely associated with tumour but also rendered CD147 less stable in the ER and more vulnerable to proteasomal degradation. Deletion of N-linked glycosylation at Asn152 significantly inhibited *in situ* tumour metastasis. Therefore, N-glycans at Asn152 appear to be critical for the CD147-mediated promotion of HCC malignant transformation.

A distinct feature of CD147 in various cells and tissues, which is based on the degree of glycosylation, is the two bands that it shows in Western blot analyses, suggesting that CD147 exists in two forms: HG-CD147 and LG-CD147^33^. Additionally, HG-CD147, which is modified with a large number of tumour-associated sugar components on the plasma membrane, is considered to be the biological functional form^24^. The results of our present study demonstrate that a site (Asn152) in the extracellular domain of CD147 is the specific site for tumour-associated glycans involved in HCC. On the one hand, deletion of N-linked glycosylation at Asn152 on CD147 was present a sharp band and retained in the ER, which formed in the non-glycosylated form. On the other hand, the removal of N-glycans at Asn152 on CD147 remarkably decreased its ability to promote HCC cell invasion and migration.

As correct folding or full assembly is not achieved even after repeated attempts, proteins that accumulate in the ER are generally identified and tagged in the protein secretory pathway by ER-resident factors and translocated to the cytoplasm where they undergo ubiquitin-dependent degradation^34^,^35^. We show here that the loss of N-glycans at Asn152 render CD147 less stable and more susceptible to degradation. The treatment of cells with MG-132 significantly restored CD147(N152Q)-EGFP protein levels, and the ubiquitination experiment also confirmed that the removal of N-glycans at Asn152 results in a pronounced increase in the ubiquitination of CD147 in the presence of MG132, indicating that after the removal of N-glycans at Asn152, CD147 were treated as misfolded or unfolded proteins and rapidly degraded in a proteasome-dependent manner in the ER. Different E3 ubiquitin ligases have their unique substrates, because of specific recognition. We demonstrated that the ubiquitin ligases Hrd1 and Gp78 mediate the ERAD of the CD147(WT)-EGFP and CD147(N152Q)-EGFP in a distinct manner. CD147(WT)-EGFP that localized to the ER was degraded mainly by Hrd1-mediated ERAD; however, CD147(N152Q)-EGFP protein was degraded directly by Hrd1- and Gp78-mediated ERAD. Furthermore, we found that under ER stress, the Hrd1 and Gp78 expression levels were up-regulated in N152Q cells compared with their expression in WT cells. The up-regulationof the ERAD system may explain why the CD147 expressed on the plasma membraneis always the highly glycosylated form (HG-CD147)^24^,^36^.

The endoplasmic reticulum contains high concentrations of molecular chaperones that help newly synthesized proteins acquire their native conformation through highly ordered steps of maturation and quality control^37^. We selectively identified four binding partners that may be responsible for regulating CD147 folding and/or degradation in the ER. Deletion of N-linked glycosylation at Asn152 on CD147, which appears to adopt an alternate conformation distinct from that of theWT, rely more strongly on a GRP78- or GRP94- dependent folding pathway. Meanwhile, under ER stress, N152Q cells preferentially increased the expression of the heat shockproteins GRP78 and GRP94 compared with those of WT cells. Based on the above results, we presumed that N-linked glycosylation at Asn152 is critical for CD147 to acquire and maintain its original and proper folding pattern in the ER. Accumulating evidences indicate that chaperones also play a role in protein degradation, which is based on the intracellular stability state of a protein^38^,^39^. We observed that ER-localized CD147 trafficked towards degradation via two phases in the retention-based ER chaperones. Inhibiting the interaction between N-glycans and CNX via treatment with CAS in WT cells caused the degradation of CD147, correlating with the transfer from CNX to GRP78. Furthermore, knockdown of both CNX and GRP78 additively delayed the degradation of CD147 in N152Q cells.

In the ER, glycans play a pivotal role in protein folding, oligomerization, quality control, sorting, and transport. They are used as a universal “tag” that allow specific lectins and modifying enzymes to establish order among the diversity of maturing glycoproteins^40^,^41^. Our data show that N-glycans at Asn152 on CD147 function as a recognition motif that is directly mediated by the CNX quality control system. The interaction between CD147 and CNX was detected in both the WT and N152Q cell lines. Meanwhile, As shown in Fig. S5, IP experiment showed that CD147 interacted with CNX in the N44/152/186Q cells, which demonstrated that there is a direct interaction between CD147 binds to CNX through protein-to-protein interaction. However, the binding between CD147 (N152Q)-EGFP and CNX was decreased relative to CNX binding with the WT, suggesting that the interaction between N-glycans at Asn152 and CNX is a dual binding system. We also observed that the inhibition of the interaction between N-glycans at Asn152 and CNX in the presence of CAS caused CD147(N44/186Q)-EGFP accumulation in the ER. Simultaneously, the association between CD147(N152Q)-EGFP and CNX was retained even after the cells were incubated with cycloheximide for 9 h compared with the observed association in the WT cells, indicating that CD147(N152Q)-EGFP had undergone repeated post-translational CNX-assisted folding attempts. Taken together, N-glycans at Asn152 appear to be crucial for CD147 to be correctly released from the CNX-mediated quality control system.

In our laboratory, we developed the specific mAb (monoclonal antibody) HAb18 against CD147 for use as animmunotherapeuticagent, which has been demonstrated to be safe and efficacious for thetargeted treatment of HCC in clinicalpractice^42^. In the present study, preventing CD147 site-specific modifications in glycosylation was shown to improve its tumour suppressive functions in HCC. Our findings potentially shed light on the molecular regulation of CD147 by glycosylation and provide a valuable (glyco) biomarker for developing drugs that target N-glycosylation at Asn152 on CD147, which may represent aneffective strategy for the early diagnosis, risk stratification, and development of therapeutic strategies for treating HCC in the clinical setting.

## Methods

### Cell culture

Human SMMC-7721 HCC cells (Institute of Biochemistry and Cell Biology, Academic Sinica, Shanghai, China) and K7721 cells (7721 CD147−/− cells, developed and preserved in our laboratory^43^) were cultured in DMEM (Gibco, New York, USA) with 10% foetal bovine serum, 2 mM glutamine, 100 U/mlpenicillin, and 100 μg/ml streptomycin in 5% CO_2_ atmosphere at 37 °C.

### Construction of stable K7721 cell lines

To obtain cell lines stably expressing wild type or mutant fusion proteins with EGFP linked to the N-terminus of the human CD147, K7721 cells were grown in 6-well plates until 80% confluence and transfected with the wild type or mutant CD147-EGFP plasmids using Lipofectamine2000 reagent (Invitrogen, Carlsbad, USA). Stable cell lines were selected by adding G-418 (at a final concentration of 0.6 mg/ml) 48 h after transfection. This concentration of G-418 was maintained until single colonies appeared. Finally, 5–9 colonies were isolated, expanded, and grown in the presence of 0.6 mg/ml of G-418 in 6-well plates.

### Antibodies and Reagents

#### Antibodies

Anti-CNX antibody (sc-80645), Anti-GFP antibody (sc-9996), Anti-Ub antibody (sc-166553), Anti-PDI antibody (sc-74551), anti-GADD153/CHOP antibody (sc-4066) and anti-α-tubulin antibody (sc-8035) were purchased from the Santa Cruz Biotechnology (Japan). Anti-CRT antibody (C7492), Anti-GRP78/Bip antibody (G9043), and Anti-GRP94 antibody (G4420) were purchased from the Sigma-Aldrich^®^ (Germany). Anti-Hrd1 antibody (14773) and Anti-GP78 antibody (9590) were from the Cell Signaling Technology (USA). Anti-GSK-3beta antibody (610201) were purchased from the BD Transduction Laboratories (USA).

#### Reagents

MG-132 (ML4834) was purchased from the Peptide Institute (Japan). Chloroquine (C6628) and castanospermine (532673) were from Sigma-Aldrich^®^ (Germany). Cyclohexinmide (EY2005) was purchased from the Amquar (USA).

### RNA interference and transfection

RNAi oligonucleotides (GenePharma,China) were as follows:

HumanCNX siRNA 5-ACACUAGUCUGUGUAACUUUA-3,

human GRP78 siRNA 5-CCAUAAGUGACACCAAUAAAUGUTT-3,

humanHrd1(#1)siRNA 5-AAUCAUCAAGGUUCUGCUGUA-3,

humanHrd1(#2)siRNA 5-AAGGTGATGGGCAAGGTGTTC-3,

humanGp78(#1)siRNA 5-AAGACGGAUUCAAGUACCUUU-3,

humanGp78(#2)siRNA5-AACGAATGCTGGTGTATAAGT-3.

Lipofectamine2000 reagent was used according to the manufacturer’s instructions. Silencer negative control siRNAwas employed as a negative control.

### Western blot analysis

Cells were harvested in lysis buffer and the total protein concentration was determined using aBCA Protein Assay Kit (Thermo Scientific, Waltham, MA). Equal amounts of protein were separated by SDS-PAGE on a 10% polyacrylamide gel and then transferred on to PVDF membranes (Millipore). After blocking with 5% non-fat milk, the membrane was incubated for 3 h at room temperature with the indicated antibody.

### Plasmids and Transfection

Plasmids for expression of CD147(WT)-EGFP, CD147(N44Q)-EGFP, CD147(N152Q)-EGFP, CD147(N186Q)-EGFP, CD147(N44/152/186Q)-EGFP, and CD147(WT)-EGFP have been described previously^24^. The human pcDNA3.1(+)-GRP78/BiP plasmid was purchased from Addgene. The detailed descriotions about plasmid can be found online (http://www.addgene.org/32701).To generate GRP78/G37T with a mutated tyrosine phosphorylation site, The QuikChange Lightning Multi Site-Directed Mutagenesis Kit was from Stratagene, Santa Clara, CA, USA. Primers used were as follows: forward primer, 5′-GTCGGCATCGACCTGACAACCACCTACTCCTG-3′, and reverse primer, 5′-CAGGAGTAGGTGGTTGTCAGGTCGATGCCGAC-3′. Cell transfections were performed using Lipofectamine 2000 according to the manufacturer’s instruction.

### Immunofluorescence staining assay

Cells were grown on chambered coverslips, fixed with 4% paraformaldehyde, permeabilized with 0.05% saponin, and then treated with specific antibodies. Cells were observed under a confocal microscope (Nikon, Tokyo, Japan).

### Cell growth tests

HCC cells were cultured in a 96-well plate (1 × 10^4^ per well) for 24 h. Each experiment was performed in triplicate. After incubation, the cells were harvested and counted every 24 h using aCCK-8 kit (Engreen biosystem, China, EC020).

### ER isolation

Isolation of the endoplasmic reticulum (ER) was performed with a Sigma endoplasmic reticulum isolation kit (Cat No: ER0100 –1KT) according to the directions of the protocol. Briefly, cells were homogenizedin icecold homogenization buffer. The homogenates were centrifuged at 14,0006 g for 20 min to remove cell debris, nuclei, and mitochondria. The supernatant wasthen subjected to a series of centrifugation steps to pellet the microsomes. The microsomal fraction wasseparated into Golgi, ER using the gradient medium Iodixanol. 50 μl fractions were collected from the top of the gradient downward with a syringe.

### Cross-linking

We cross-linked proteins in the cell using the cell-permeable, thiol-cleavable primary amine crosslinker dithiobis (DSP; Thermo FisherScientific, Madison, WI, USA). Cells were treated with DSP at a final concentration of 2 mM for30 min at room temperature, followed by the quenching of the crosslinker with an excess of Tris-HCl, pH 7.5, for 15 min at room temperature. After cross-linking, cells were lysed in an IP buffer supplemented with protease inhibitors.

### Co-immunoprecipitation

Co-immunoprecipitation (co-IP) was performed using a Thermo Scientific Pierce co-IP kit (Cat: 26149) according to the manufacturer’s protocol. K7721 cells stably expressing CD147(WT)-EGFP or CD147(N152Q)-EGFP were separately used. Briefly, the antibody was first immobilized for 3 h using AminoLink Plus coupling resin. The resin was washed and incubated overnight with arterial lysate. After incubation, the resin was again washed and the protein was eluted using an elution buffer. A negative control that was provided with the IP kit to assess non specific binding was treated in the same manner as the co-IP samples, including the addition of the antibody. In this control, the coupling resin was not amine-reactive, preventing the covalent immobilization of the primary antibody onto the resin. Samples were analysed by Western blotting. The control resin provides an excellent negative control when processed the same as the Antibody Coupling resin. IP antibody: Hrd1 (H7915, Sigma), anti-Gp78 (9590, Cell Signalling Technology, USA), CD147-EGFP (sc-9996, Santa Cruz, Japan).

### Mass Spectrometry Analyses

To analyze IP protein samples by ESI-MS (Electrospray IonizationMass Spectrometry) in the positive ion mode, samples were evaporated and reconstituted in 1% (v/v) formicacid, followed by trypsin digestion (Promega) prior to injection into the mass spectrometer. The mass spectrometer (Model LTQ, Thermo Fisher Scientific) was operated in a data-dependent MS/MS mode in which the instrument cycled between full MS scans (m/z 300–2000) and intervening MS/MS scans on the ten most intense ions occurring in the MS scan. The acquired MS/MS spectra were searched using the Mascot protein database search program (Matrix Science) against a full database of human protein sequences. The detailed descriotions of proteins were further confirmed can be found online (http://www.addgene.org/32701).

### *In vitro* invasion assays

The assay was performed using chambers with polycarbonate filters (8 μM pore size; Millipore). The upper side of a polycarbonate filter was either coated or not coated with Matrigel to form a continuous thin layer. The HCC cells (1 × 10^5^) were resuspended in 300 μL of 0.1% serum-free medium and added to the upper chamber. The lower chamber was filled with 10% FBS medium (200 μL). After a 24-h incubation, the filters were fixed with methanol for 10 min and stained with 0.5% methylrosaniline chloride for 20 min. The cells on the upper surfaces of the filters were removed with a cotton swab. The cells on the reverse sides were counted in five random fields of view using a microscope at a 100× magnification. Each assay was performed in triplicate.

### Wound healing assay

Cells (2 × 10^6^) were plated in 6-well plates and cultured to approximately 100% confluence. The cells were scraped with a pipette tip and rinsed to remove non-adherent cells, Then, fresh medium with 1% foetal bovine serum was added. The cells were rinsed with 10% FBS medium for 24 h and then examined using a phase contrast microscope (Olympus, Japan, Tokyo).

### Nude mouse xenograft studies

Male BALB/c nu/nu mice (4 weeks of age and 16–20 g) were provided by the Laboratory Animal Research Center of FMMU, and the animal study was reviewed and approved by the FMMU Animal Care and Use Committee. The experiment was carried out in accordance with the relevant guidelines^44^. Briefly, The mice were housed in a standard animal laboratory under constant environmental conditions. A mixture of K7721 cells stably expressing CD147(WT)-EGFP or CD147(N152Q)-EGFP (1 × 10^6^) in 0.1 ml culture medium and the same volume of diluted Matrigel were subcutaneously injected (100 μl per mouse) the flank of each mouse. Mice were randomly divided into two groups, each containing 4 mice. The length (L) and width (W) of tumors were measured with vernier caliper every 4 days. Tumor volume (V) was calculated with the formula V = (L × W^2^) × 0.5. On the 20th day after injection, mice were sacrificed and tumors were harvested and photographed.

### Establishment of the orthotopic transplant nude mouse model of HCC metastasis

For the *in vivo* metastasis assays, experimental metastatic potential was assessed following as described in detail previously^45^,^46^. Briefly, A mixture of K7721 cells stably expressing CD147(WT)-EGFP or CD147(N152Q)-EGFP (1 × 10^6^) in 0.1 ml culture medium and the same volume of diluted Matrigel was injected into the left liver lobe of the nude mice. Mice were randomly divided into two groups, each containing 4 mice. The mice were sacrificed 8 weeks after implantation. The number of intrahepatic metastases was calculated and statistically analyzed. Livers and lungs were then were dissected, fixed in formalin, embedded in paraffin. Hematoxylin-eosin staining staining was performed, and the sections were examined by a pathologist to verify the presence of metastatic nodules.

### Haematoxylin-eosin-stain(HE)

Thin slices of tumor tissue for all cases were fixed in 4% formaldehyde solutionfor periods not exceeding 24 h. The tissues wereprocessed for paraffin embedding, and 4 mm-thicksections were cut and stained with hematoxylin and eosin. Stained sections were observed and digital images were taken with a light microscope (Olympus BX-53, Tokyo, Japan).

### Statistical analysis

Data were analysed using GraphPad Prism 6 (GraphPad Software, Inc.), and results are expressed as the means ± standard error of the mean. Two-tailed Student’s *t*-tests were used to evaluate significance. Statistical significance was set at *P < 0.05, **P < 0.01, and **P < 0.001.

## Additional Information

**How to cite this article**: Li, J.-H. *et al*. N-linked glycosylation at Asn152 on CD147 affect protein folding and stability: promoting tumor metastasis in hepatocellular carcinoma. *Sci. Rep*. **6**, 35210; doi: 10.1038/srep35210 (2016).

**Publisher’s note**: Springer Nature remains neutral with regard to jurisdictional claims in published maps and institutional affiliations.

## Supplementary Material

Supplementary Information
