# Supplementary material for: N-linked glycosylation at Asn152 on CD147 affects protein folding and stability: promoting tumour metastasis in hepatocellular carcinoma
Source: Sci Rep. 2016 Nov 21;6:35210. doi: 10.1038/srep35210 (PMC5116672; doi:10.1038/srep35210)
Supplement: Supplementary Information [file srep35210-s1.doc]

**N-linked glycosylation at Asn152 on CD147 affects protein folding and stability: promoting tumour metastasis in hepatocellular carcinoma**

Jiang-Hua Li*, Wan Huang*, Peng Lin*, Bo Wu*, Zhi-Guang Fu^1^, Hao-Miao Shen^1^, Lin Jing^1^,Zhen-Yu Liu^1^, Yang Zhou^1^,Yao Meng^1^, Bao-Qing Xu^1^, Zhi-Nan Chen*^1^,Jian-Li Jiang*^1^

Cell Engineering Research Centre &

Department of Cell Biology

Fourth Military Medical University

No.169, Changle West Road, Xi’an, China

**Supplemental information**


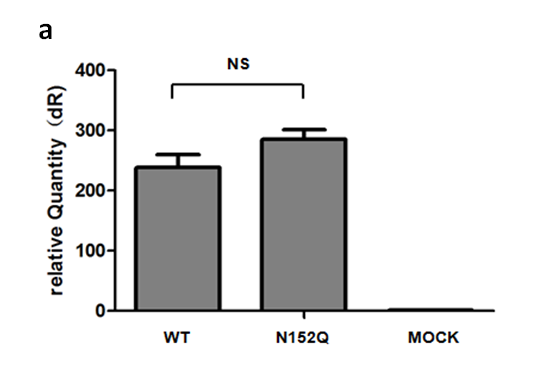


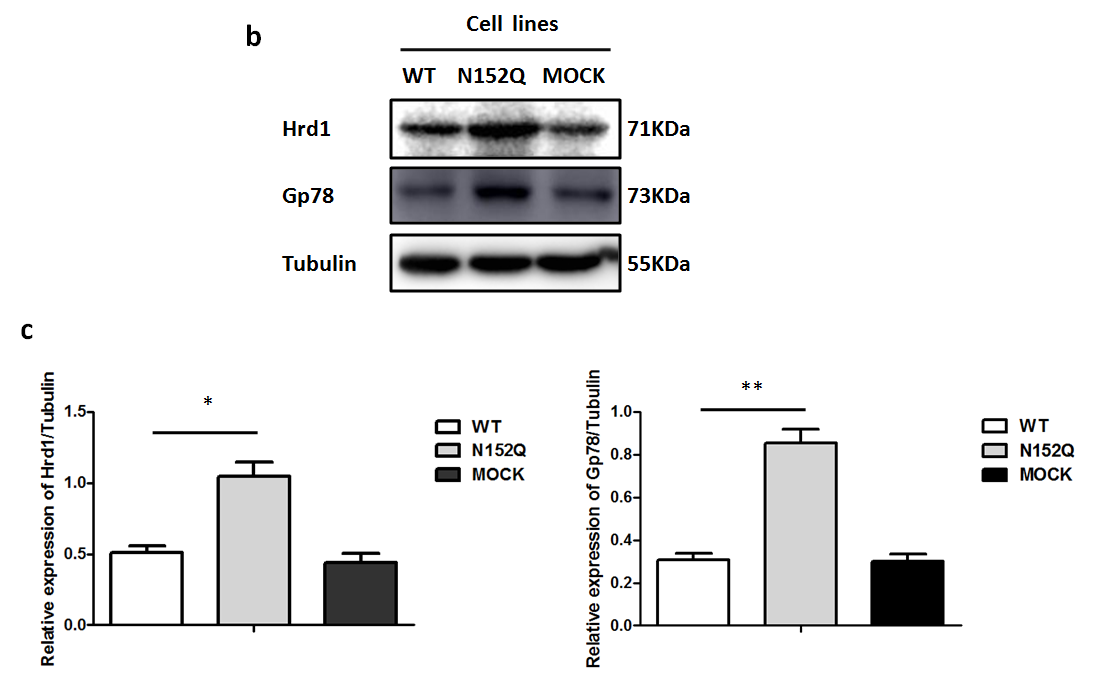


Figure S1: (a) the level of mRNA in WT and N152Q cell lines. Real-time RT-PCR analysed CD147 of mRNA level in the WT and N152Q cells. (b) Western blot analysis of Hrd1 and Gp78 expression in the WT and N152Q cells. (c) Quantitative analysis of Hrd1 and GP78 protein expression in each cell line. Values indicate the mean ± standard error of the mean (SEM) of three independent experiments. **P*< 0.05, ***P*< 0.01.


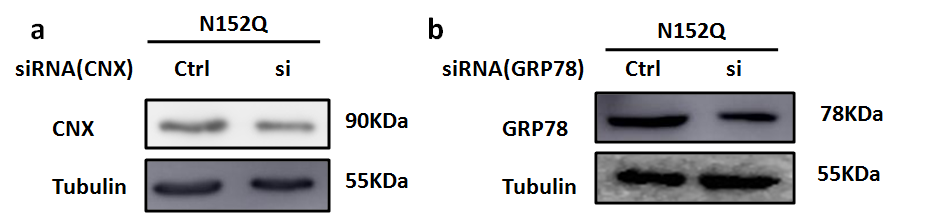


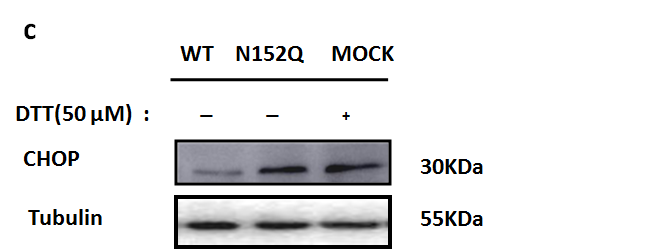


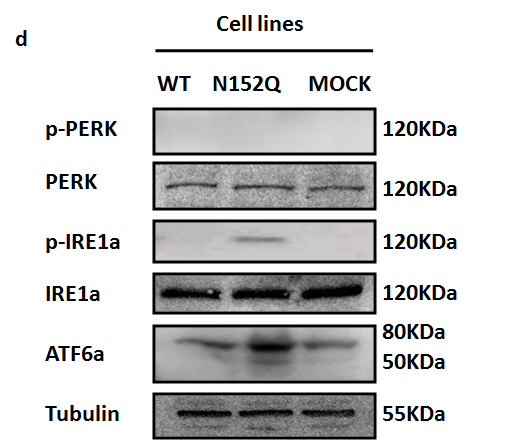


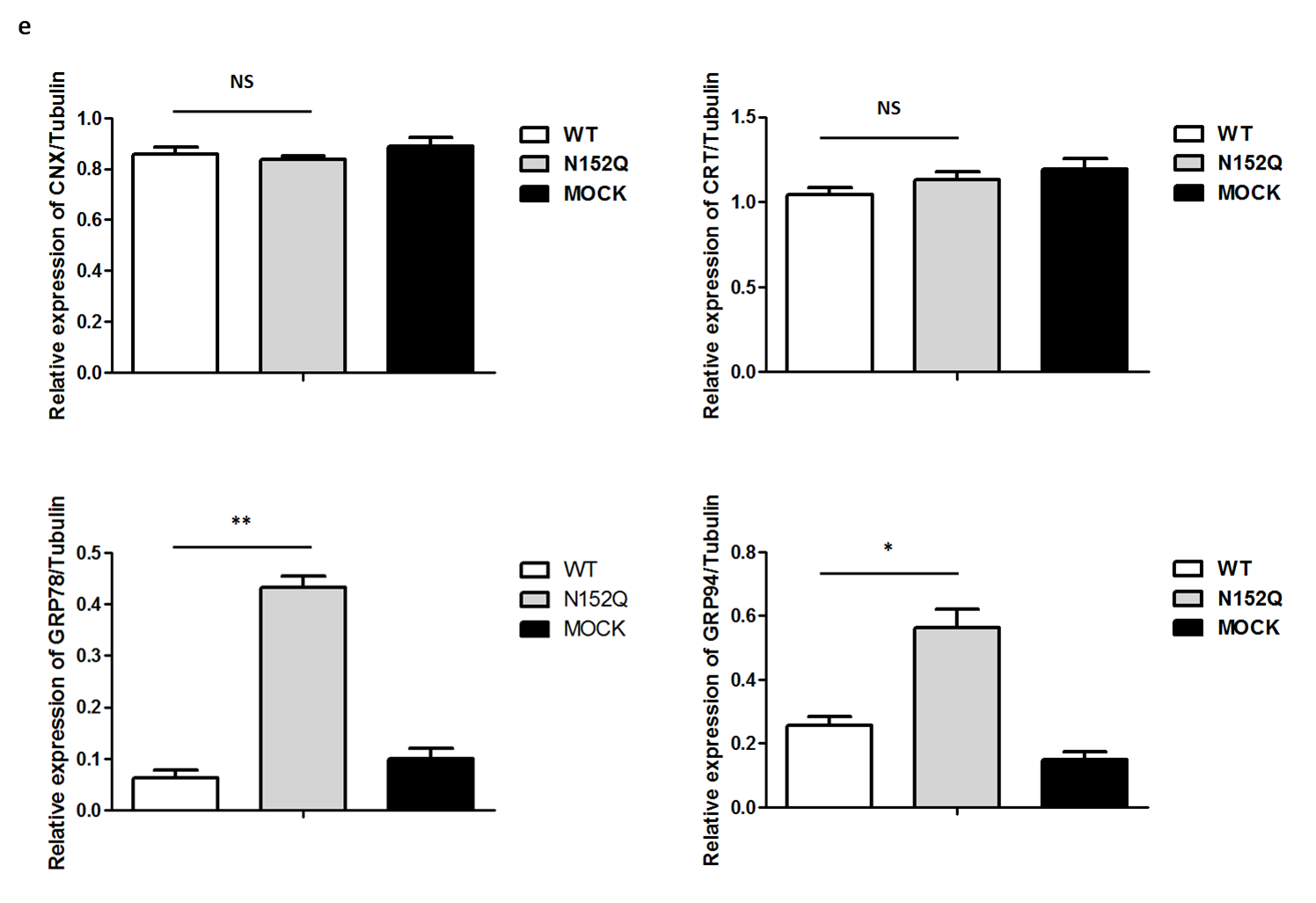


Figure S2: (a) Knockdown of CNX. K7721 cells stably expressing CD147(N152Q)-EGFP were transfected with control or CNX siRNAs for 3 days. Immunoblots of cell lysates were probed with the indicated antibodies. (b) Knockdown of GRP78. K7721 cells stably expressing CD147(N152Q)-EGFP were transfected with control or GRP78 siRNAs for 3 days. Immunoblots of cell lysates were probed with the indicated antibodies. (c) ER stress. K7721 cells stably expressing CD147(WT)-EGFP or CD147(N152Q)-EGFP were analysed by Western blotting to determine the levels of the pro-apoptotic ER stress indicator protein CHOP. Cells in the presence of 50μM DTT for 24 h were used as an ER stress-induced control. (d) UPR analysis. Western blot analysis of PERK, p-PERK, IRE1a, p-IRE1a, ATF6a expression in the WT and N152Q cells.(e) Quantitative analysis of CNX, CRT,GRP78 and GRP94 protein expression in each cell line. Values indicate the mean ± standard error of the mean (SEM) of three independent experiments. no statistical significance (NS), **P*< 0.05, ***P*< 0.01.


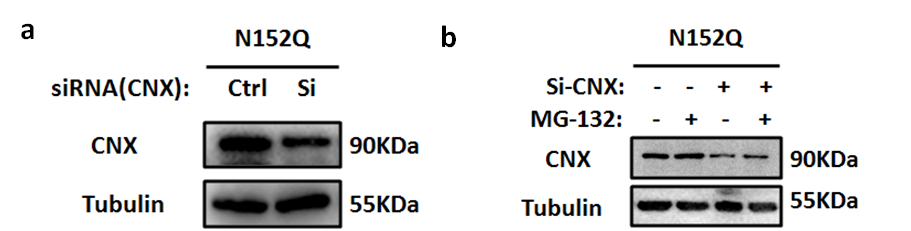


Figure S3: (a and b) Knockdown of CNX. K7721 cells stably expressing CD147(N152Q)-EGFP were transfected with control or CNX siRNAs for 3 days. Immunoblots of cell lysates were probed with the indicated antibodies.


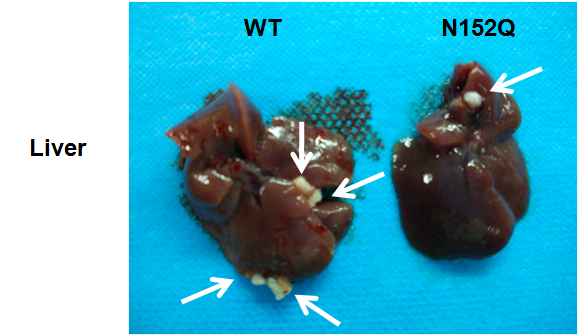


Figure S4: Metastasis assay by intrahepatic injection of K7721 cells stably expressing CD147(WT)-EGFP or CD147(N152Q)-EGFP cell lines in nude mice. Liver were excised for examination. Intrahepatic metastases in each group, as determined by calculation of metastatic lesions in the whole liver (n= 4).


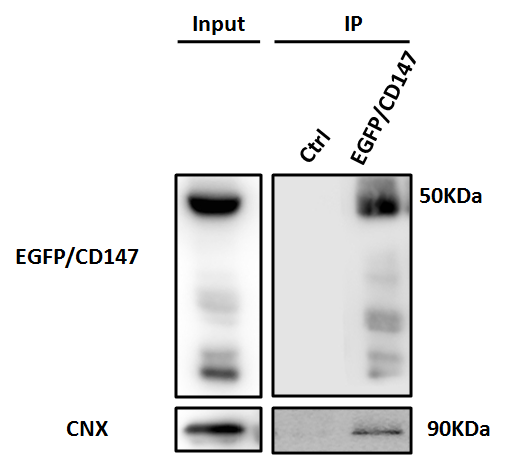


Figure S5: CD147(N44/152/186Q)-EGFP immunoprecipitated with CNX. Extracts prepared from K7721 cells stably expressing CD147(N44/152/186Q)-EGFP were immunoprecipitated using the indicated antibodies. The resulting precipitates were examined by immunoblot analysis using the indicated antibodies.

Table S1:   Identification of chaperons by LC-MS/MS in Crosslinking experiments


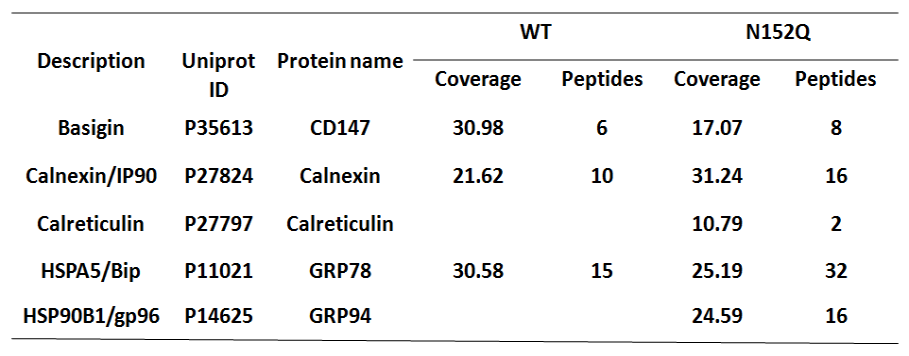


**Methods**

**To screen and identify the CD147(WT)-EGFPand CD147(N152Q)-EGFP interactors.**

To screen proteins that interact with CD147(WT)-EGFP and CD147(N152Q)-EGFP. K7721cells stably expressing CD147(WT)-EGFP or CD147(N152Q)-EGFP were separately used. In view of the fact that the interaction between chaperones and target protein is a transient, weak interaction, it is difficult to perform a comprehensive and accurate screening in the ER. Thus, DSP (a thiol-cleavable, homobifunctional and amine-reactive crosslinking agent) was used in the cross-linking experiment to make the interaction between CD147 and binding partners bond covalently. This experiment can help us find more binding partner. Briefly, cells were treated with DSP at a final concentration of 2mM for 30min at room temperature, followed by the quenching of the crosslinker with an excess of Tris-HCl, pH 7.5, for 15 min at room temperature. Notably，The number of cells should be as many as possible and time gradient should be added. After cross-linking, cells were lysed in an IP buffer supplemented with protease inhibitors. Next，IP experiment was used to find CD147(N152Q)-EGFP binding partners. In this article, EGFP antibody was used to prey the CD147-EGFP protein and captured proteins. Co-immunoprecipitation (co-IP) was performed using a Thermo Scientific Pierce co-IP kit (26149) according to the manufacturer's protocol. Briefly, the EGFP antibody was first immobilized for 3 h using AminoLink Plus coupling resin. The resin was washed and incubated overnight with arterial lysate. After incubation, the resin was again washed and the protein was eluted using an elution buffer. A negative control that was provided with the IP kit to assess nonspecific binding was treated in the same manner as the co-IP samples, including the addition of the EGFP antibody. In this control, the coupling resin was not amine-reactive, preventing the covalent immobilization of the primary antibody onto the resin. Samples were analysed by Western blotting. At last, to identify IP protein samples by ESI-MS (Electrospray IonizationMass Spectrometry) in the positive ion mode, samples were evaporated and reconstituted in 1% (v/v) formicacid, followed by trypsin digestion (Promega) prior to injection into the mass spectrometer. The mass spectrometer (Model LTQ, Thermo Fisher Scientific) was operated in a data-dependent MS/MS mode in which the instrument cycled between full MS scans (m/z 300-2000) and intervening MS/MS scans on the ten most intense ions occurring in the MS scan. The acquired MS/MS spectra were searched using the Mascot protein database search program (Matrix Science) against a full database of human protein sequences. The detailed descriotions of captured proteins were further confirmed can be found online (<http://www.addgene.org/32701>).
